# Amygdalin: Toxicity, Anticancer Activity and Analytical Procedures for Its Determination in Plant Seeds

**DOI:** 10.3390/molecules26082253

**Published:** 2021-04-13

**Authors:** Ewa Jaszczak-Wilke, Żaneta Polkowska, Marek Koprowski, Krzysztof Owsianik, Alyson E. Mitchell, Piotr Bałczewski

**Affiliations:** 1 Department of Analytical Chemistry, Faculty of Chemistry, Gdansk University of Technology, 11/12 Narutowicza Str., 80-233 Gdansk, Poland; ewajaszc@student.pg.edu.pl; 2 Division of Organic Chemistry, Centre of Molecular and Macromolecular Studies, Polish Academy of Sciences, Sienkiewicza 112, 90-363 Łódź, Poland; mkopr@cbmm.lodz.pl (M.K.); owsianik@cbmm.lodz.pl (K.O.); 3 Department of Food Science and Technology, University of California, Davis, One Shields Avenue, Davis, CA 95616, USA; aemitchell@ucdavis.edu; 4 Institute of Chemistry, Faculty of Science and Technology, Jan Długosz University in Częstochowa, Armii Krajowej 13/15, 42-200 Częstochowa, Poland

**Keywords:** amygdalin, hydrogen cyanide, cyanogenic glycosides, analytical procedures, almond, anticancer activity, toxicity

## Abstract

Amygdalin (d-Mandelonitrile 6-*O*-β-d-glucosido-β-d-glucoside) is a natural cyanogenic glycoside occurring in the seeds of some edible plants, such as bitter almonds and peaches. It is a medically interesting but controversial compound as it has anticancer activity on one hand and can be toxic via enzymatic degradation and production of hydrogen cyanide on the other hand. Despite numerous contributions on cancer cell lines, the clinical evidence for the anticancer activity of amygdalin is not fully confirmed. Moreover, high dose exposures to amygdalin can produce cyanide toxicity. The aim of this review is to present the current state of knowledge on the sources, toxicity and anticancer properties of amygdalin, and analytical methods for its determination in plant seeds.

## 1. Introduction 

Diseases related to industrial civilization are one of the biggest problems in developing and highly developed countries. Technological progress and the resulting environmental pollution are related to the increase in rates of diseases, such as cancer, diabetes, osteoporosis, overweight as well as cardiovascular, neurodegenerative and autoimmune diseases [1]. Cancer is a group of diseases involving unregulated cell growth with the potential to invade or spread to other tissues in the body [2]. In general, about 5–10% of cancers can be attributed to genetic defects whereas 90–95% are attributed to environment and lifestyle including smoking, diet (fried foods, red meat), obesity, low physical activity, excessive alcohol consumption, sun exposure, environmental pollution, infections and stress [3]. Although cancer is largely considered a preventable disease, the number of cancer-related deaths continues to increase worldwide [2]. In response, the World Health Organization (WHO) has increased campaigns focusing on research, early detection and prevention in order to identify life-style changes and medical interventions for the treatment of cancer [4]. At present, there are about 400,000 people in Poland diagnosed with various types of cancer [5] and more than 10 million people worldwide [2].

The most common medical approaches for treating cancer include surgical procedures, radiotherapy, chemotherapy, as well as several methods that are often used simultaneously to achieve a synergistic effect. The most common alternative approaches include modified diets, acupuncture, hypnotherapy and bioenergotherapy as well as the use of natural products including amygdalin [6,7]. 

**Figure 1:**
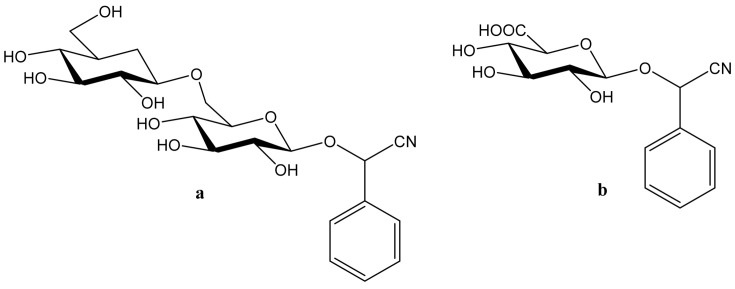
Chemical structures of amygdalin (**a**) and Laetrile (**b**).

Amygdalin (d-Mandelonitrile 6-*O*-β-d-glucosido-β-d-glucoside) is a naturally occurring disaccharide, a source of HCN, highly concentrated in fruit kernels from *Rosaceae* species, for example, in bitter almonds, apricot and peach [1]. Bitter almonds have been used since ancient times to treat fevers, headache (via their purging activity) and as a diuretic [8]. Amygdalin is composed of two molecules of glucose, benzaldehyde and hydrogen cyanide and can exist in the form of two *R* and *S* epimers (Figure 1a) [9]. *R*-Amygdalin is natural amygdalin and *S*-amygdalin is called neoamygdalin.

**Figure 2:**
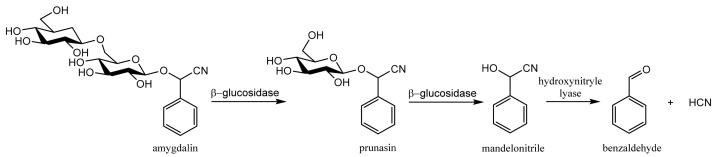
Hydrogen cyanide formation as a result of hydrolysis of amygdalin.

Beta-glucosidase stored in compartments of plant cells is also present in the human small intestine [10] and degrades amygdalin into prunasin, mandelonitrile, glucose, benzaldehyde and hydrogen cyanide (Figure 2). Hydrogen cyanide (HCN), benzaldehyde, prunasin and mandelonitrile, can be absorbed into the lymph and portal circulations [11]. The anticancer activity of amygdalin is thought to be related to the cytotoxic effects of enzymatically released HCN and non-hydrolyzed cyanogenic glycosides [12].

Laetrile (d-Mandelonitrile-β-glucuronide), which is derived from amygdalin, has been used as a complementary and alternative natural medicine (CAM) in the treatment of cancer for over 30 years [13] (Figure 1b). Studies of amygdalin on various cancer cell lines demonstrated their anticancer activity [14], but the statements related to a patient study, made by the U.S. Food and Drug Administration (FDA) in the late 1970s [15] did not confirm this. Since then, however, many publications have been presented confirming both the toxicity occurring with excessive consumption of amygdalin contained in bitter almonds and the therapeutic, especially anticancer, properties of amygdalin [16]. Many papers have also been published describing methods for the determination of amygdalin in food products, which is of crucial importance in the context of the ambivalent effects of these compound. Therefore, the aim of this review is to present the current state of knowledge on the sources and toxicity of cyanogenic glycosides and analytical methods for determination of amygdalin in plant seeds [2].

**Figure 3:**
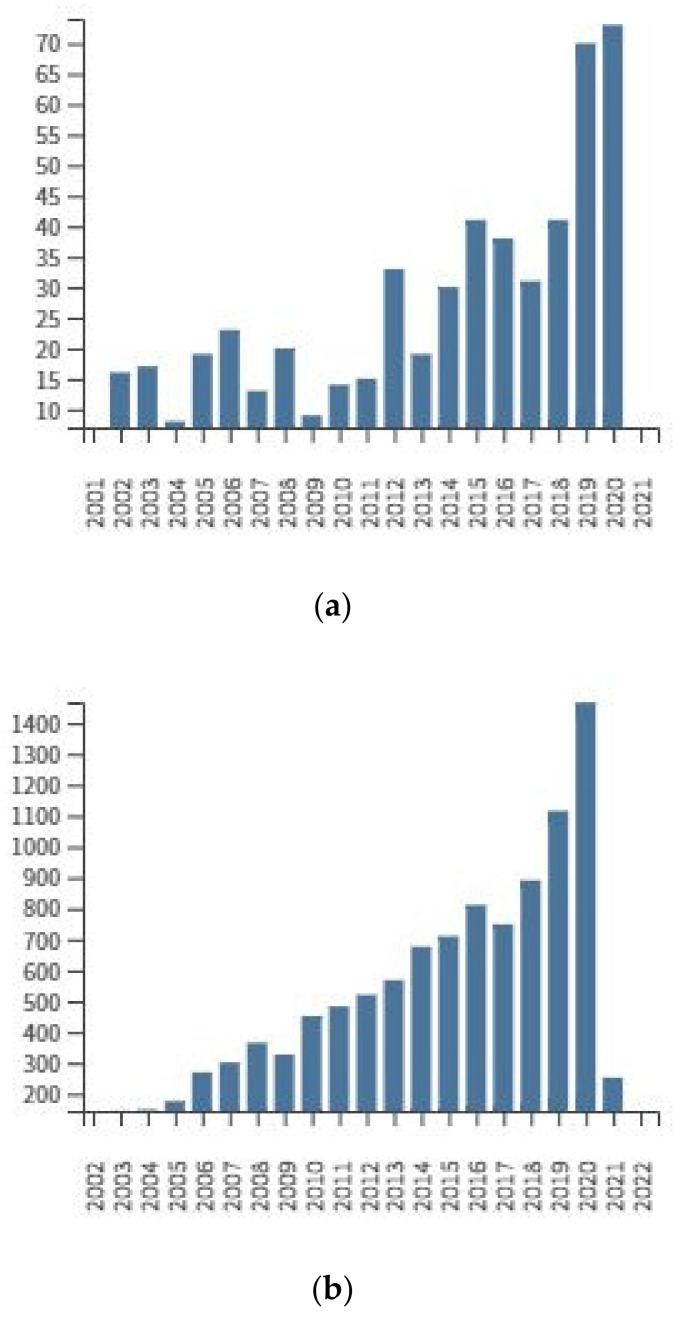
Total publications by year (**a**) and sum of times cited by year (**b**) for amygdalin as a topic (Web of Science^®^, accessed on 3rd March 2021).

Growing interest in the biological activity of amygdaline and related research problems (Figure 3) are difficult to estimate accurately due to the many similar keywords that are simultaneously or alternatively used in literature of the subject. According to the Web of Science^®^ database (Accessed on: 24 March 2021), the number of hits for individual entries was: vitamin B17 (26), Laetrile (315), amygdalin (725), cyanogenic glycoside (957). The following number of quotations have been found for the descriptor of amygdalin and: analytical procedures (14), anticancer (26), toxicity (83), almond (93), cancer (123), seeds (156).

## 2. Amygdalin as a Member of Cyanogenic Glycosides—Their Sources, Toxicity and Anticancer Activity

Amygdalin belongs to the cyanogenic glycosides (CGs), which are group of organic chemical compounds composed of sugar(s) and an aglycon containing 1-cyanobenzyl moiety. The 1-cyanobenzyl moiety is linked to the hemiacetal OH group located at the anomeric carbon atom of the sugar moiety (Table 1). CGs can be classified not only as cyanohydrin derivatives where OH group is functionalized with sugar moiety, but also as a group of organic cyanides (nitriles) of the RCN type. Sometimes, nitriles are also included in the group of pseudohalogenes [17]. To the group of primary CGs belong also: prunasin, linamarin, dhurrin, vicianin, prulaursin, sambunigrin, neolinustatin, taxifylline, lotaustralin and linustatin [18].

### 2.1. Sources

Cyanogenic glycosides-containing plants occur in about 2000 species belonging to 110 families (e.g., Rosaceae, Poaceae, Papilionaceae, Euphorbiaceae, Scrophulariaceae), including many plants and seeds of edible fruits, such as peach and edible kernels of almond (Table 2). The natural function of cyanogenic glycosides is to protect the plant against insects and larger herbivores [19]. The content of amygdalin usually increases during the fruit enlargement stage and remains constant or minimally decreases during ripening. In the peach seed, the amygdalin content is greater in the endocarp than in the mesocarp. Bitterness in the almond kernel is determined by the content of the cyanogenic amygdalin diglucoside [20]. 

**Figure 4:**
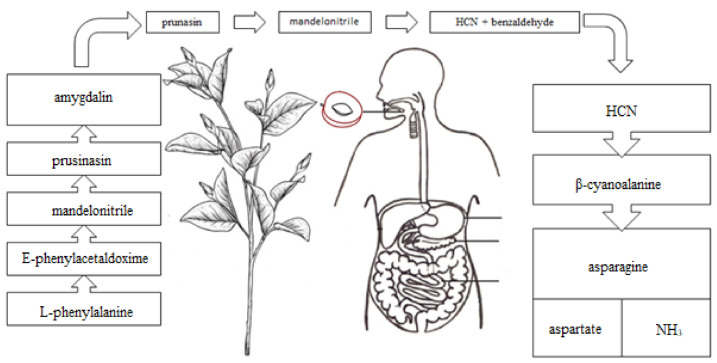
Hydrogen cyanide formation as a result of hydrolysis of amygdalin.

Biosynthesis of amygdalin involves the initial conversion of L-phenylalanine into mandelonitrile catalyzed by cytochrome P450 and CYP71AN24. By the action of UDP-glucosyltransferase, mandelonitrile is converted to prunasin. The glucosyltransferase catalyzes conversion of prunasin into amygdalin [22]. Plants containing CGs usually contain degradation enzymes, such as β-glycosidases (E.C. 3.2.1.21) which hydrolyze α-glucosidic bonds and lead to formation of α-hydroxy nitriles (cyanohydrins) and sugar moieties. Hydroxynitrile lyases (E.C. 4.1.2.47) catalyze further dissociation of cyanohydrins to carbonyl compounds (benzaldehyde) and hydrogen cyanide (Figure 4). The HCN release occurs when tissues of a cyanogenic plant are macerated, e.g., when eaten by herbivores, resulting in contact of CGs with enzymes that hydrolyze them. These enzymes can be deactivated by thermal denaturation (e.g., hot water, high temperature). In plants that do not have β-glycosidase enzymes, but contain CGs, hydrolysis can be achieved in the digestive tract of animals and humans, provided that gastrointestinal endosymbiotes produce β-glycosidase [18]. In humans, the decisive formation of HCN is probably caused by the bacterial flora of the intestine that is able to produce β-glycosidase in the brush border of the small intestine [10,12,23].

### 2.2. Toxicity

Compounds with the CN group, both of the organic (RCN) and inorganic (HCN, CN-anions) origin are absorbed into the body through the gastrointestinal tract, as well as through the respiratory system and skin. They lead to inactivation of enzymes containing ferric ions (Fe^3+^). For example, a key enzyme of the respiratory chain-cytochrome oxidase, binding to the active site of cytochrome c oxidase, inhibits oxygen metabolism, especially in myocardium and brain cells [24]. In animals, hydrogen cyanide reacts with methemoglobin in the blood, but most cyanide metabolism occurs in tissues [25]. A significant (80%) part of cyanides is detoxified in liver. This is due to a thiosulfate sulfur-transferase enzyme (i.e., rhodanase [E.C. 2.8.1.1]), which is present in the liver mitochondria. Sulfur, which is necessary for this reaction, is taken from biological compounds, e.g., thiosulfates. Rhodanase transforms cyanides into thiocyanates, which are quickly excreted in urine. The process of the cyanide metabolism in the living organism may take place in various ways (Figure 3). One example is combining cyanide with hydroxocobalamin (vitamin B12a), to obtain cyanocobalamin (i.e., vitamin B12). The remaining cyanide ions are oxidized to formates and carbon dioxide. Formates are excreted in urine and carbon dioxide, where together with hydrocyanic acid are excreted through the lungs. A small amount of cyanides also combine with cysteine to form 2-iminothiazolidine-4-carboxylic acid [26]. 

The toxic dose of hydrogen cyanide released by enzymatic hydrolysis of CGs in plant tissues is defined as the dose exceeding 20 mg of hydrogen cyanide per 100 g of fresh weight [27]. Excessive consumption of seeds may have a negative effect on the body, causing a number of adverse reactions of the following types: diarrhea, vomiting, abdominal pain and in extreme cases may lead to death (Table 3). Human lethal dose of intravenous injection of amygdalin is 5 g [28]. No data are available for other fruits of Poland’s climate zone. It is believed that the consumption of 50 bitter almonds in a short period of time can be a lethal dose for an adult and that a dose of 5–10 bitter almonds can be poisonous for a child. The adult lethal dose of amygdalin is estimated to be 0.5–3.5 mg/kg body weight [1,29]. 

### 2.3. The Anticancer and Other Biological Activities

CGs medical applications are mainly related to amygdalin, discovered in 1830 by French chemists Pierre-Jean Robiquet and Antoine François Boutron-Charlard. A theory by Dr. Ernst T. Krebs, Sr., that amygdalin could be an effective drug against cancer, but is too toxic for humans, was announced in 1920. Despite this statement, his son Ernst Theodore Krebs, Jr., synthesized in 1952 a less harmful amygdalin derivative with one subunit of glucose, which he called Laetrile [34]. The mixture of amygdalin and its modified form was described by Krebs as “vitamin B17” [35,36] although in the literal sense neither amygdalin nor Laetrile are vitamins. In 1977, the FDA (USA) issued a statement indicating that there was no evidence of the Laetrile safety and efficacy [2]. 

While it is forbidden to sell amygdalin and Laetrile in the U.S. and Europe, there are laboratories and clinics in Mexico offering amygdalin preparations and therapies for many years (e.g., Cyto Pharma De Mexico, 40 years on the market) [37]. However, there is no solid clinical data to support the efficacy of these therapies on patients [38]. In contrast, in vitro cell culture studies show, a number of amygdalin activities that would be beneficial in cancer treatment (Table 4). For example, amygdalin has the capacity to control apoptotic proteins and signaling molecules, which may be a justification for a decrease in tumor proliferation. Amygdalin treatment increased expression of Bax, decreased expression of Bcl-2 and induced caspase-3 activation in human DU145 and LNCaP prostate cancer cells [9], induced apoptosis of HeLa cervical cancer cells mediated by endogenous mitochondrial pathway [39] and reduced adhesion and migration of UMUC-3 and RT112 bladder cancer cells through activation of focal adhesion kinase (FAK) and modulation of β1 integrin [40]. Amygdalin has also the ability to inhibit anti-apoptotic expression of genes including Survivin, and XIAP genes [13]. Other biological activities of amygdalin have also been demonstrated and they include antibacterial [41,42,43], antioxidant [44,45], anti-atherosclerotic [46], anti-asthmatic [47], preventing lung [48] and liver fibrosis [49]. Amygdalin also improves microcirculatory disturbance, attenuates pancreatic fibrosis [50], possesses anti-inflammatory and analgesic activity [51], stimulates muscle cell growth [39] and finally may serve as a beneficial agent in treating a dry eye disease [52]. 

## 3. Determination of Amygdalin in Plant Seeds

### 3.1. Collection, Transport and Storage of Plant Seeds

The first steps of any analytical procedure are sampling, transport and storage of the material for further analysis. If these steps are not performed properly, the time and cost and value of the analysis may be increased or limited. Additionally, samples may degrade or change, and incorrect chemical identification and quantification errors may occur. Typically, all fruits, vegetables and food products should be obtained using a logically thought out random sampling plan, collected and stabilized (e.g., freezing, refrigeration, drying, etc.) as soon as possible.

The maturation state of the fruit should be defined at the time of harvest and aligned with the analytical goals (e.g., determining a specific state of maturation or commercial maturity). Transportation to the laboratory should be under controlled or defined conditions (e.g., refrigeration). For the CG analysis, a fruit should be separated into peel, flesh and kernel. The fruit can be dried at room temperature [56] or lyophilized [57]. The plant material is next fragmented and homogenized using a mortar [58] or by blender [59] and sieved to a specific and defined particle size. In case of almonds, the skins is removed by blanching (i.e., immersion in hot water) [60]. Controlling the storage conditions is critical for maintaining the integrity of the samples prior to extraction and analysis. Samples are usually stored at −80 °C until they are analyzed to inhibit enzymatic degradation [56,61]. 

### 3.2. Sample Preparation

**Figure 5:**
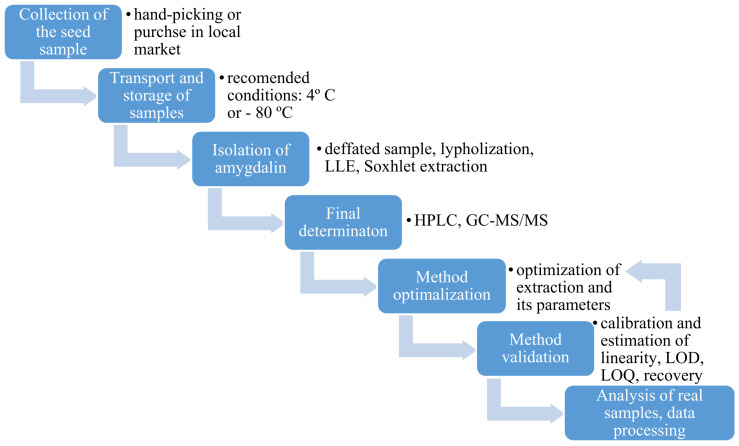
General workflow during the analysis of seed samples.

Seed samples have a complex matrix, therefore they may require additional preparation for analysis (Figure 5). Many problems are associated with the fatty matrix and the low concentrations of compounds present in the samples. Most samples will require multiple extraction steps with both aqueous and organic solvents. The solubility of amygdalin in water and ethanol is 83 g·L^−1^ and 1 g·L^−1^, respectively. In water, amygdalin hydrolyzes into benzaldehyde, hydrocyanic acid, glucose [59] and can be converted into *S*-amygdalin (neoamygdalin), during extraction, refluxing and/or storage making it ineffective against cancer [6] Amygdalin is easily hydrolyzed by acids and bases, so control of pH is critical. Amygdalin epimerization occurs in boiling water and especially under alkaline conditions due to the weakly acidic character of the benzyl proton. It is also important to note that due to the tendency of amygdalin to epimerize at higher temperatures, extractions should be performed at temperatures lower than 100 °C [62].

When fruit samples are collected, their amount is expressed in kg, but after preparation of a suitable representative sample the amount needed for analysis can be 0.1 to 5 g. After fruit sample collection and inactivation of enzymes, samples are homogenized and a suitable extraction solvent is selected. Amygdalin is extracted from seeds with polar solvents, such as ethanol, methanol, ethyl acetate and water. It has been observed that polar solvents give low amygdalin recovery due to conversion of the natural isomer into the *S*-amygdalin. In the presence of water and weak bases, the epimerization of stereogenic carbon occurs and *S*-amygdalin is formed. Neoamygdalin may also be transformed into amygdalin during processing [63,64,65]. 

If the seed samples contain a lot of fat, one may consider use of diethyl ether [19], diethyl ether [66] or n-hexane [67] to remove the fat first without losses in amygdalin and other CGs. Organic solvents are removed through drying before aqueous extractions are performed. In order to minimize the amount of the solvents used and improve extraction efficiency, dynamic extraction can be performed with refluxing using a Soxhlet extractor [19,56]. The efficiency of a static extraction can be improved with an ultrasonic bath [68]. The most widely used extraction method for amygdalin is the solid-phase extraction (SPE) using a C_18_ extraction column [69,70,71]. To avoid the epimerization of amygdalin, extractions are performed at temperatures lower than 100 °C, usually at 35–40 °C [59,66,68]. While amygdalin is easily soluble in methanol and ethanol, it can also be extracted into water containing 0.1% citric acid under reflux, which may be a more environmentally green option [58]. Finally, the obtained supernatant is filtered through cartridge and/or syringe filter, and the diluted sample is analyzed. 

### 3.3. Analytical Techniques for Plant Seeds Analysis

After sample preparation, the next step in a general analytical procedure is to select the relevant analytical technique for the final determination. The choice depends on several factors, such as concentration of analytes in the sample, composition of the matrix and the concentration of interfering agents. Raman spectroscopy or FT-IR can be used to check the cyanogenic glycoside distribution in the fruit stone sample because organic compounds and functional groups can be identified by their unique vibrational pattern [72]. The presence of broad peaks at 3150–3600 cm^−1^ represents the stretching vibration of OH groups in the amygdalin structure. Aliphatic C-H stretching vibrations and the vibrations of aromatic ring appear at 2885–2927 cm^−1^. Amygdalin can be probed by the Raman spectroscopy due to a characteristic band of the nitrile group at 2245 cm^−1^ in a part of the spectrum that is free from interference of frequencies due to other chemicals. The peaks at 1620 cm^−1^ and 864 cm^−1^ are due to aromatic C=C and aromatic C-H bending, respectively [72,73]. Raman linear mapping studies on bitter almond seeds showed that the amygdalin content increased from the seed center to the margin [74]. In apricot seeds, amygdalin is unevenly distributed and its location does not follow the same pattern for all seeds [75].

A review of literature shows that amygdalin is measured in plant seeds samples primarily using high-performance liquid chromatography (HPLC) [56,66,67] and gas chromatography (GC) methods [76,77]. However, HPLC is more convenient than gas chromatography because of its ability to separate overlapping signals and eliminate background. The primary type of chromatographic column used to resolve amygdalin in seed samples is C_18_ which is filled with ultra-pure silica modified with low polarity octadecyl groups. The predominant detectors used in the HPLC technique include UV–Vis [60], diode array (DAD) [68], mass spectrometry (MS) [58] and MS/MS ones [78]. The HPLC-MS identification of cyanogenic glucosides is usually performed in the positive ionization mode. The SIM MS spectrum of amygdalin contains a peak at *m/z* 458 corresponding to the [M+H]^+^ ion of amygdalin. It is possible to eliminate any potential false positives, by monitoring the 475–325 transitions [22]. However, in MS/MS cases, negative ionization resulted in a better sensitivity as compared to the positive ionization. Quantification of amygdalin was achieved using the transition of 456–323 [79]. The purity and structural identification of the extracted amygdalin was verified spectroscopically using the UV–Vis technique [80,81]. The presence of signal peaks at wavelengths of 1370–1400 nm indicates the O-H stretching modes of water absorption, while regions of 1100–1600 nm and 1700–2300 nm corresponded to sugar display bands [60]. The maximum absorption of amygdalin was detected at 214 nm using a photodiode array detector [66]. However, the use of methanol in separation of amygdalin from the extract may have an unsatisfactory resolution [81]. Moreover, NMR spectroscopy was used for additional structural characteristics of amygdalin. The ^1^H-NMR and ^13^C-NMR nuclear resonances of amygdalin diluted in DMSO-d_6_ was performed [73]. A summary of the recent literature on analytical procedures for determination of amygdalin in seed samples is summarized in Table 5.

## 4. Future Trends and Conclusions

**Figure 6:**
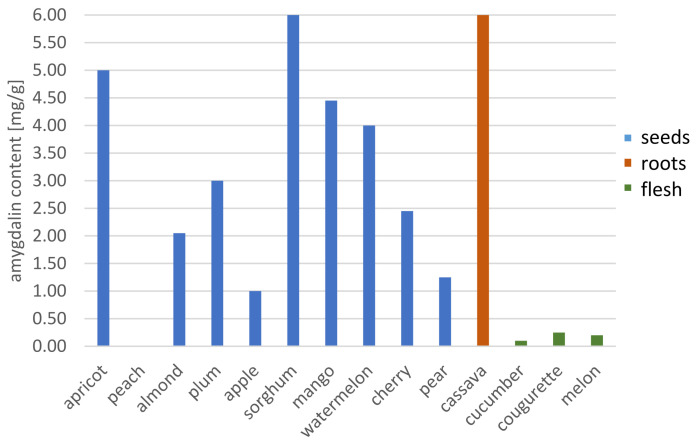
Changes in the amygdalin content in edible plants.

Research on cyanogenic glycosides has increased dramatically over the past 10 years. Much of this interest centers on the cytotoxic effect of amygdalin on cancer cells in vitro and understanding the distribution of amygdalin in plants that are commonly consumed in the human diet. The amygdalin content of various edible plants differs depending on the kind and the region where they are cultivated (Figure 6). As amygdalin is synthesized in response to environmental stress, agronomic and environmental factors such as latitude, climate and variety of the plant can influence its levels in plant tissues. Understanding the distribution and levels of cyanogenic glycosides in plants is important as poisoning with cyanides have usually occurred by accident and due to a lack of awareness of levels in a food or natural products. To date, there have been numerous cases of cyanide poisoning resulting from the ingestion of too many seeds containing amygdalin and as the result of the Laetrile treatment, but there are no reports of people cured of cancer by consuming the seeds containing amygdalin.

Research into the use of amygdalin in the treatment of cancer continues. There is evidence confirming the cytotoxic effect of amygdalin on cancer cells in vitro, however, these results are not yet demonstrated in clinical studies. Nevertheless, there is still a need for quantifying the levels of amygdalin and other CGs in plant materials in order to support the clinical trials, and to better understand their intake in the human diet. New studies on amygdalin isolation and characterization should employ principles of green chemistry, and eliminate the use of toxic solvents, such as methanol. Moreover, novel spectrophotometric techniques can be explored for determining CG content in real time (e.g., FTIR spectroscopy).

## Figures and Tables

**Table 1:** Information on selected cyanogenic glycosides.

Cyanogenic Glycoside		Occurrence	Ref
Amygdalin	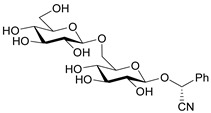	*Amygdalus communis*, *Cydonia oblonga*, *Padus*	[3]
Prunasin	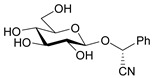	*Prunus*	[4]
Vicianin	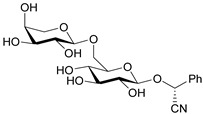	*Papilionaceae*	[5]
Linmarin	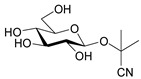	*Linum, Phaseolus*	
Sambunigryn	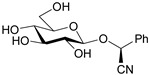	*Sambucus*	

**Table 2:** Concentration of hydrogen cyanide released in the process of enzymatic hydrolysis of amygdalin in various parts of plant tissues [21].

Plant		Cyanogenic Potential [mg HCN/kg Plant Material]
Peach	Kernel	710
Plum	Kernel	696
Nectarine	Kernel	196
Apricot	Kernel	785
Apple	Seed	690

**Table 3:** Information on amygdalin poisoning.

Patient	Dose	Effects	Ref
Child (2 years)	500 mg	vomiting, apathy, diarrhea, accelerated breathing	[30]
Child (4 year)	500 mg	diarrhea, accelerated breath, blood cyanide concentration 163 µg/L	[31]
Adult woman (80 years)	300 mL	dyspnea, vertigo and vomiting.	[32]
Adult woman	9 g	vomiting, dizziness, blood cyanide concentration 143 µg/L	[33]

**Table 4:** Examples of in vitro cytotoxicity studies on cancer cells.

Cell Lines Used for Testing		Amygdalin Concentration [mg mL^−1^]	Results Observed	Ref.
Bladder cancer	RT 112 UMUC-3 TCCSUP	1.25–10	limited proliferative capacity and apoptosis. decrease in cdk4 expression level in RT112 and TCCSUP lines.	[40]
Cervical cancer	HeLa	1.25–20	initiation of the cell apoptosis, reduction of Bcl-2 expression level, increase of Bax expression level.	[39]
Colon cancer	SNU-C4	0.25–5	reduction of the expression level of many genes associated with following cell functions: growth, apoptosis, transmission.	[53]
Breast cancer	MDA-MB-231, MCF-7	2.5–80	reduction of proliferative activity of the cells	[54]
	MDA-MB-231	10	growth rate of cancer cells was inhibited	[55]
Kidney cancer	Caki-1 A498 KTC-26 xds	10	-reduced ability collagen and fibronectin. -reduced cell mobility.	[47]

**Table 5:** Total amygdalin content in different samples.

Analytical Technique	Sample	Recovery [%]	Intra/Inter-Day Variation [%]	LOD	LOQ	Detected Compounds in Real Samples	Ref
LC-DAD	apricot seeds	91 ± 10	0.8/3.8	1.2 mg·L^−1^	4.0 mg·L^−1^	bitter seeds 26 ± 14 mg·g^−1^ sweet seeds 0.16 ± 0.09 mg·g^−1^	[82]
	apricot liqueur	-	-	-	-	38.79 µg·mL^−^^1^	[83]
	cherry liqueur					16.08 µg·mL^−^^1^	
HPLC–MS/MS	almonds	-	-	200 µg·g^−^^1^	-	<LOD	[22]
HPLC-UV	plum seeds	-	-	-	-	25.30 g 100 g^−^^1^	[59]
	almonds	-	0.13/0.75	2 µg·mL^−^^1^	-	4.51 ± 0.04%	
	loquat fruit kernel	-	-	-	-	7.58 ± 0.76 mg·g^−1^	
	almonds	98.4–102.9	0.25/0.31	0.02 mg·L^−1^	0.07 mg·L^-1^	sweet: <350 mg·kg^−1^ bitter: 14,700–50,400 mg·kg^−1^	[60]
	peach seeds	99.05	0.19	0.03 mg 100 g^−^^1^	0.09 mg 100 g^−^^1^	6.3 ± 0.2 g 100 g^−^^1^	[84]
	plum seeds					0.439 ± 0.001 g 100 g^−^^1^	
	apricot seeds					7.9 ± 0.2 g 100 g^−^^1^	
	peach seeds	-	-	-	-	seed: 12.14 ± 4.80 mg 100g^−^^1^	[19]
	citrullus colocynth kernels	97.34 ± 0.58	-	0.88 mg·L^−^^1^	2.93 mg·L^−^^1^	0.27 ± 0.03 100 g^−^^1^	[72]
	apples	-	0.095	0.0505 mg·g^−^^1^	0.0548 mg·g^−^^1^	0.28–1.40 mg·g^−^^1^	[73]
	Armeniacae semen	98.0–102.6	-	-	-	45.42 ± 1.21 mg·g^−1^	[85]
	bitter almond oil	96.0–102.0	4.8/7.2	0.07 µg·mL^−1^	-	0.092 ± 0.003 mg·g^−1^	[86]
	wild almond oil	-	-	-	-	12.8–12.9 mg/100 mL oil	[87]
	sweet apricot kernels	-	-	-	-	5.0 ± 0.23 mg·g^−^^1^	[88]
HPLC-DAD	apricot seeds	-	-	-	-	0.861 g·100 g^−1^	[56]
	almonds	-	-	-	-	0.37–1.46 g·kg^−^^1^	[89]
	apple seeds	98	-	0.1 µg·mL^−^^1^	-	1–3.9 mg·g^−^^1^	[66]
	apricots	-	-	0.1 µg·mL^−^^1^	0.3 µg·mL^-^	14.37 ± 0.28 mg·g^−^^1^	[67]
	cherries					2.68 ± 0.02 mg·g^−^^1^	
	peaches					6.81 ± 0.02 mg·g^−^^1^	
	pears					1.29 ± 0.04 mg·g^−^^1^	
	cucumbers					0.07 ± 0.02 mg·g^−^^1^	
	courgettes					0.21 ± 0.13 mg·g^−^^1^	
	melons					0.12 ± 0.07 mg·g^−^^1^	
	apricot kernels	-	-	0.2 µg·mL^−^^1^	-	-	[69]
	apricot kernels	99.08	2.4/3.5	-	-	0.217–0.284 mg·mL^−^^1^	[58]
	plum seeds	-	-	1.06 μg·mL^−^^1^	3.49 μg·mL^−^^1^	25.30 g 100 g^−^^1^	[65]
	bayberry kernels	77.9	-	-	-	129.13–358.68 mg·L^−^^1^	[57]
	food suplements	94.81	0.57/1.52	0.13 mg·L^−1^	0.40 mg·L^−^^1^	20.68 ± 1.58 mg·g^−1^	[86]
	apricot kernels	91	0.8/3.8	1.2 mg·L^−1^	4.0 mg·L^−1^	26 ± 14 mg·g^−1^	[90]
UHPLC-(ESI)QqQ MS/MS	nonbitter almonds	-	-	0.1 ng·mL^−^^1^	0.33 ng·mL^−1^	63.13 ± 57.54 mg·kg^−1^	[64]
	semibitter almonds					992.24 ± 513.04 mg·kg^−1^	
	bitter almonds					40,060.34 ± 7855.26 mg·kg^−1^	
	alomnds	-	-	-	-	1.62–76.50 mg·kg^−^^1^	[67]
Spectrophotometric method	cassava root	-	-	-	-	3.40 mg·L^−^^1^	[80]
	cassava roots	-	-	-	-	8.84–48.33 mg·g^−^^1^	[91]
	sorghum seeds					122.31 mg·g^−^^1^	
	mango seeds					4.41 mg·g^−^^1^	
	watermelon seeds					3.97 mg·g^−^^1^	
	almond seeds					3.91 mg·g^−^^1^	
ELISA	black cherries	99 ± 1.2	-	200 ± 0.05 pg·mL^−^^1^	-	2.14 ± 0.15 mg·g^−^^1^	[92]
	yellow plums					2.30 ± 0.90 mg·g^−^^1^	
	peaches					5.79 ± 0.83 mg·g^−^^1^	
	black plums					9.75 ± 1.32 mg·g^−^^1^	

- no data.

## Data Availability

No new data were created or analyzed in this study. Data sharing is not applicable to this article.

## Sample Availability

Samples of the compounds are not available from the authors.
